# Algorithm for transient response of human open‐circuit whole‐room indirect calorimetry: A review and future perspective

**DOI:** 10.14814/phy2.70624

**Published:** 2025-10-22

**Authors:** Insung Park, Hitomi Ogata, Kumpei Tokuyama

**Affiliations:** ^1^ Institute of Global Well‐being Science Hirosaki University Graduate School of Medicine Hirosaki Japan; ^2^ Graduate School of Humanities and Social Sciences Hiroshima University Hirosaki Japan; ^3^ International Institute for Integrative Sleep Medicine University of Tsukuba Tsukuba Japan

**Keywords:** whole‐room indirect calorimetry, time resolution, deconvolution

## Abstract

Open‐circuit whole‐room indirect calorimetry is a reliable method for assessing cumulative energy expenditure and substrate oxidation over a prolonged period without disrupting normal activities such as eating and sleeping. Over the past three decades, efforts to improve the time resolution of indirect calorimetry have increased, aiming to synchronize energy metabolism with rapidly changing behaviors and physiologic signals. As such, various new algorithms have been proposed: trend identification, total variation denoising, wavelet de‐noising, Kalman estimation, and deconvolution with a regularization parameter. The ability to estimate the time course of energy metabolism differs among algorithms. In this review on the methodology of open‐circuit whole‐room indirect calorimetry, we discuss similarities and critical differences in the theoretical background of various algorithms and compare their performance in simulations. In terms of correlation with the true value and mean squared errors, deconvolution with a regularization parameter outperformed the other algorithms. As further improvements in the algorithmic approaches are expected, we propose a practical guideline to support ongoing development: newly developed algorithms should be benchmarked against existing algorithms, and the source code should be made publicly available to facilitate transparent evaluation and future comparisons.

## Graphical abstract

Whole‐room indirect calorimetry has been a reliable method for assessing cumulative energy expenditure and substrate oxidation over a prolonged period, that is, 24 h. Improving time resolution of the system with deconvolution with a regularization parameter, it is now able to measure time course of energy metabolism.

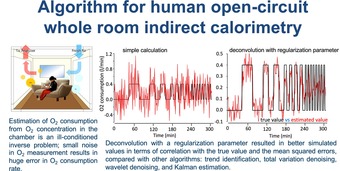

## 1 INTRODUCTION

The theoretical basis of gaseous exchange in open‐circuit whole‐room indirect calorimetry was described by Brown et al. (1984). Symbols used throughout this review are consistent with those employed by Brown et al. (1984). (Table 1). Currently, more than 40 research laboratories on four continents house whole‐room indirect calorimeters for human studies (Chen et al., 2020), with the main research target of whole‐room indirect calorimetry being cumulative energy expenditure and substrate oxidation over 24 h. Over the past three decades, efforts to improve the time resolution of indirect calorimetry have increased, aiming to synchronize energy metabolism with rapidly changing behaviors and physiologic signals. Energy expenditure increases after a meal is consumed (thermic effect of food, TEF), and the physiologic significance of this effect on weight gain has been studied (Tataranni et al., 1995). Quantifying the TEF magnitude requires an understanding of the temporal relationship between energy expenditure and body movement to isolate activity thermogenesis from total energy expenditure (Purcell et al., 2024). In addition to the magnitude of TEF, the rate of changes in energy expenditure and substrate oxidation after meal intake provides valuable information regarding human metabolic flexibility (Carnero et al., 2021).

**TABLE 1:** List of symbols.

Variables
V: volume of metabolic chamber [L]
F: flow rate [L/min]
f: fractional concentration of oxygen [%]
R: rate of oxygen consumption [L/min]
t: time [min]
Subscripts
i: incoming
o: outgoing
Regularization factors
λ: for total variation denoising
γ: for deconvolution

*Note*: In this review, gas volumes are expressed under standard temperature, pressure, and dry conditions (STPD). The conversion factor to STPD condition (ψ) has been omitted from all equations for better readability. Discussion on methodological issues focused on oxygen consumption as an example, with the subscript “G”, denoting “any gas”, omitted from all equations. In simulations, the respiratory quotient was set at 1.0, that is, F_o_ = F_i_ and the Haldane factor does not appear in equations.

The limited time resolution of whole‐room indirect calorimetry is mainly due to the dilution of expired gas with the large volume of ambient air in the chamber. As a result, rapid changes in O_2_ consumption rate are attenuated in the measured O_2_ concentration in the chamber. On the other hand, when the O_2_ consumption rate is estimated from changes in the O_2_ concentration in the chamber, the whole‐room indirect calorimetry system behaves as a high‐pass filter, amplifying the high‐frequency components of the gas production rate. This review provides an overview of algorithms aimed at improving the time resolution of whole‐room indirect calorimetry while suppressing irregularities in the estimated time course of O_2_ consumption. This review is organized as follows. Section 2 summarizes the classical theory of open‐circuit whole‐room indirect calorimetry. The highly irregular time course of the O_2_ consumption rate is mainly due to an amplified error in time derivatives of O_2_ volume in the chamber (d/dt(Vf_o_)). Section 3 evaluates algorithms developed to suppress noise in d/dt(Vf_o_), including trend identification (Henning et al., 1996), total variation denoising (Chen et al., 2018), and wavelet de‐noising (Brychta et al., 2009). In Section 4, as a different stochastic approach to suppress highly irregular O_2_ consumption rate in whole‐room indirect calorimetry, deconvolution with a regularization parameter (Tokuyama et al., 2009) and Kalman estimation (Granato et al., 2004) are introduced. In Section 5, we compare the performance of various algorithms in simulations. Algorithms reviewed in Sections 3 and 4 are found scattered in the literature from various disciplines, sometimes without cross‐reference. In Sections 3, 5, theoretical similarities and critical differences among algorithms are discussed. Section 6 presents criticisms of deconvolution with a regularization parameter and simulations. In Section 7, physiologic implications of newly developed algorithms for whole‐room indirect calorimetry are discussed. Finally, in Section 8, as further improvements in the algorithms are anticipated, we propose a practical guideline to facilitate future development. Aiming to enhance the readability of this methodologic review while at the same time promoting scholarly evaluation of the presented algorithms, we have omitted some details of the algorithms in the text. Some of the technical details are provided in the Appendix, and the source code for the deconvolution approach was uploaded to GitHub.

## 2 CLASSICAL THEORY OF OPEN‐CIRCUIT WHOLE‐ROOM INDIRECT CALORIMETRY

The oxygen consumption rate (R) of the subject in a metabolic chamber is related to the amount of incoming O_2_ (F_i_f_i_), outgoing O_2_ (F_o_f_o_), and the rate of changes in the O_2_ volume in the chamber (d/dt(Vf_o_)) (1).

(1) $$R=F_{i}f_{i}-F_{o}f_{o}-d/\mathrm{dt}\left({\mathrm{Vf}}_{o}\right)$$

**FIGURE 1:**
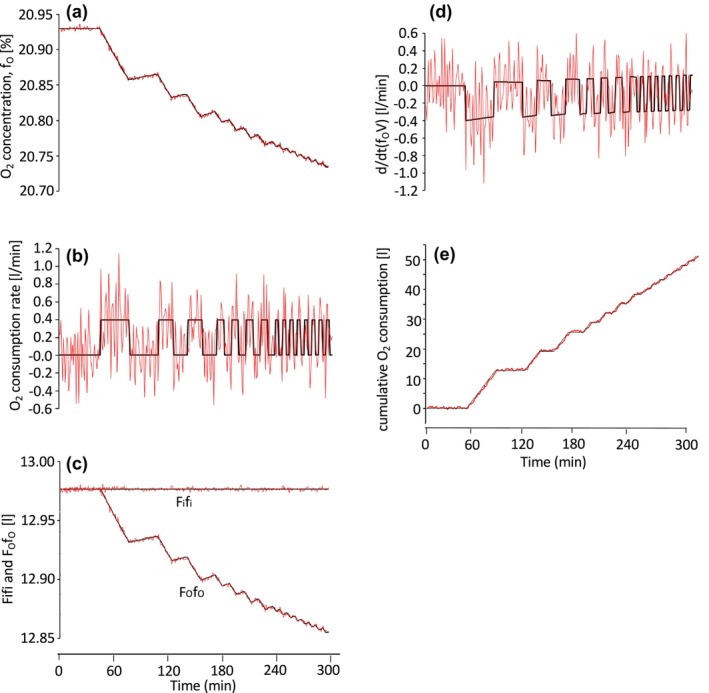
Simulation with a rectangular input signal. For simulations, the chamber of the whole‐room calorimeter was modeled as a single compartment (V = 16,626 L), from which air was pulled out at a rate of 62 L/min. O_2_ and CO_2_ concentrations of incoming air were 20.93% and 0.03%, respectively. At 45 min into the simulation, O_2_ consumption with a 64‐min period (32 min on, 32 min off) was initiated, followed by period of 32‐, 16‐, and 8‐min durations. (a) The outgoing air O_2_ concentration was calculated at 1‐min intervals (**―**) and corrupted with white noise (0.000 ± 0.002%, ―). (b–d) Estimates of O_2_ consumption rate is calculated by Equation 1 (b), from incoming (F_i_f_i_) and outgoing O_2_ volume (F_o_f_o_) (c), and time derivative of O_2_ volume in the chamber d/dt(f_o_V) (d). (e) Cumulative O_2_ consumption are shown for true value (**―**) and for noise‐corrupted data (―).

**FIGURE 2:**
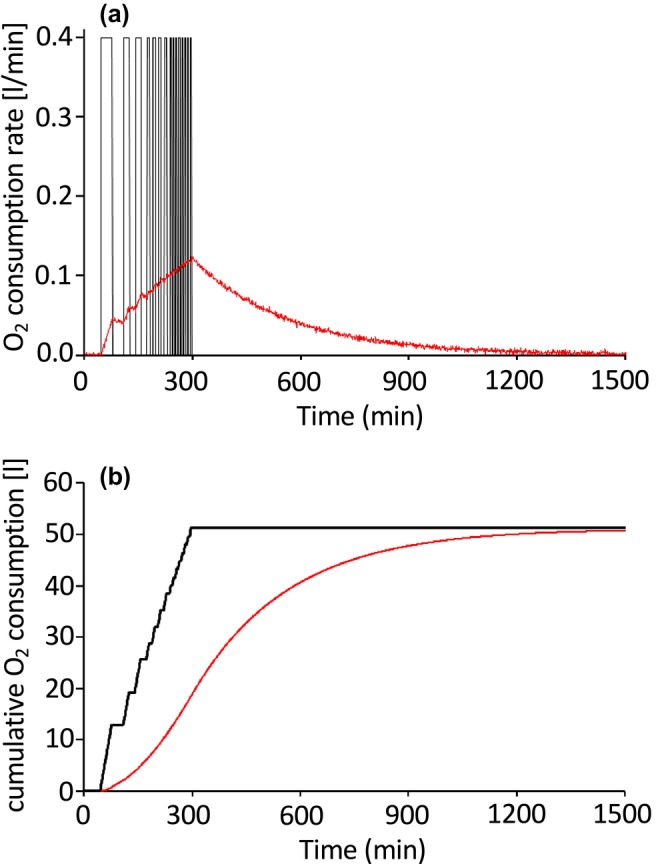
Omission of d/dt(Vfo) from Equation 1. For simulations, the chamber of the whole‐room calorimeter was modeled as a single compartment (V = 16,626 L), from which air was pulled out at a rate of 62 L/min. O_2_ and CO_2_ concentrations of incoming air were 20.93% and 0.03%, respectively. At 45 min into the simulation, O_2_ consumption with a 64‐min period (32 min on, 32 min off) was initiated, followed by period of 32‐, 16‐, and 8‐min durations. From 301 to 1500 min, O_2_ consumption rate was set at 0 L/min. The outgoing air O_2_ concentration was calculated at 1 min intervals (**―**) and corrupted with white noise (0.000 ± 0.002%). (a) True O_2_ consumption rate (**―**) was compared with that calculated as F_o_f_o_ − F_i_f_i_ from noise‐corrupted data (―). (b) Cumulative O_2_ consumption was calculated for true value (**―**) and noise‐corrupted data (―).

The contribution of F_i_f_i_, F_o_f_o_, and d/dt(Vf_o_) to R assessed in a simulation is shown in Figure 1. Intermittently repeated bouts of O_2_ consumption were pre‐determined as true values. The concentration of O_2_ in the metabolic chamber was calculated every minute and subsequently corrupted with artificially generated noise (mean ± SD: 0.000 ± 0.002%) (Figure 1a). From the noise‐corrupted O_2_ concentration, the O_2_ consumption rate was calculated using Equation 1 and compared against the true values. The effects of noise in gas concentration measurements have a distinct impact on O_2_ kinetics in open‐circuit whole‐room indirect calorimetry. First, small measurement noise in the O_2_ concentration leads to a huge irregularity in the O_2_ consumption rate—referred to as an ill‐posed problem (Figure 1b). Second, among the three terms determining the O_2_ consumption rate in Equation 1, variability in d/dt(Vf_o_) is considered to be the main component contributing to a highly irregular O_2_ consumption rate (Figure 1c,d). In this simulation, d/dt(f_o_) was calculated as a 3‐min central difference method; the f_o_ value at minute n‐1 was subtracted from the f_o_ value at minute n + 1, yielding d/dt(f_o_) at min n (Brychta et al., 2009). Evaluating d/dt(f_o_) over shorter time intervals increases the magnitude of error, an inherent trade‐off for achieving higher time resolution in open‐circuit indirect calorimetry. Furthermore, irregularities in d/dt(f_o_) are amplified by a large chamber volume (V), which is more than two orders of magnitude greater than the volume of air exchanged per minute (i.e., the flow rate, F). Third, cumulative O_2_ consumption over an extended period remains a robust estimate, even in the presence of noise in O_2_ concentration measurements (Figure 1e). Fourth, as an exact equation for the gas production rate, Brown et al. (1984) included time derivatives of the gas volume in the chamber (d/dt(f_o_V)) in Equation 1 (1). If d/dt(f_o_V) is omitted from Equation 1 to suppress irregularity in the estimated O_2_ consumption rate (R), as proposed previously (Dauncey et al., 1978), the profile of the O_2_ consumption rate is skewed, that is, the magnitude of its peak is underestimated and the time course is delayed (Figure 2a). It is important to note, however, that cumulative O_2_ consumption asymptotically approaches the true value (Figure 2b). Open‐circuit whole‐room indirect calorimetry is a reliable method to estimate cumulative energy expenditure and substrate oxidation over a prolonged period.

## 3 ALGORITHMS TO SUPPRESS IRREGULARITY IN d/dt(f_O_)

As a main component leading to irregularity in the O_2_ consumption rate, variability in d/dt(Vf_o_) was targeted in three algorithms: trend identification (Henning et al., 1996), total variation denoising (Chen et al., 2018), and wavelet de‐noising (Brychta et al., 2009). Trend identification is the most popular among similar approaches. In addition to the original algorithm for push‐type whole‐room calorimetry presented by Henning et al. (1996), an algorithm for a pull‐type system was proposed (Nguyen et al., 2003). This algorithm fits two connected exponential lines of variable length to each gas concentration measurement for the preceding 30‐min period. Regardless of the location of the intersection between the two lines, the gas concentration (f_o_) and its time derivative (d/dt(f_o_)) are evaluated at 15 min based on one of the exponential lines. The process is repeated every minute, and the newly computed O_2_ concentration and its time derivative are substituted in Equation 1. This algorithm instantaneously responds to a change from one steady state of O_2_ consumption to another, but it computes peculiar results when changes in O_2_ consumption occur more than twice within 30 min (see Figure 6b).

**FIGURE 3:**
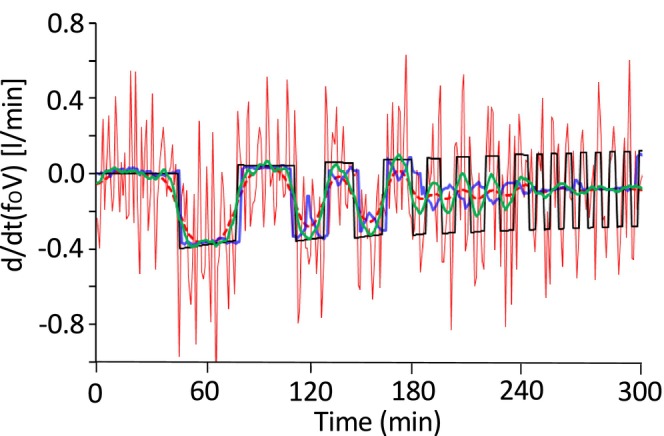
Algorithms to suppress irregularity in d/dt(f_o_). For simulations, the chamber of the whole‐room calorimeter was modeled as a single compartment (V = 16,626 L), from which air was pulled out at a rate of 62 L/min. O_2_ and CO_2_ concentrations of incoming air were 20.93% and 0.03%, respectively. At 45 min into the simulation, O_2_ consumption with a 64‐min period (32 min on, 32 min off) was initiated, followed by period of 32‐, 16‐, and 8‐min durations. The outgoing air O_2_ concentration was calculated at 1 min intervals (**―**) and corrupted with white noise (0.000 ± 0.002%). In this simulation, d/dt(f_o_) was calculated as a 3‐min central difference method; the f_o_ value at minute n‐1 was subtracted from the f_o_ value at minute *n* + 1, yielding d/dt(f_o_) at min *n* (Brychta et al., 2009). Irregularities in d/dt(f_o_V) (true values: **―**) become pronounced in the presence of measurement noise (―). High irregularity in d/dt(f_o_V) is suppressed by trend identification (**―**) (Henning et al., 1996), total variation denoising (**‐ ‐**) (Chen et al., 2018), and wavelet de‐noising (**―**) (Brychta et al., 2009). To calculate d/dt(f_o_) during the first and last 15 min of the simulation by trend identification, the simulation was extended for 15 min before and after 300 min. During the additional 15‐min periods, the O_2_ consumption rate was set at 0.00 L/min.

In another effort to suppress irregularity in d/dt(f_o_), a total variation denoising method was introduced (Chen et al., 2018).

(2) $$\text{argmin}\left\{\frac{1}{2}\sum_{n=1}^{N}{\left|y\left[n\right]-x\left[n\right]\right|}^{2}+\lambda \sum_{n=1}^{N‐1}\left|x[n+1]‐x[n]\right|\right\}$$

where *y*[*n*] is d/dt(f_o_) calculated from successive gas concentration measurements as a 3‐min central difference, and *x*[*n*] is an unknown value of d/dt(f_o_). The first term measures the fidelity to the data as the sum of squared residuals, and the second term is introduced to penalize the roughness of the estimate. The relative weight given to data fit and solution regularity is governed by the regularization parameter λ. When λ is too large, d/dt(f_o_) is overdamped, and when the values are too small the estimates for d/dt(f_o_) are highly irregular, that is, overfitting. The total variation denoising method chooses λ, which minimizes the sum of fidelity to the data and roughness of the estimate. An alternative approach to suppress the roughness of the d/dt(f_o_) is wavelet de‐noising (Brychta et al., 2009). Calculated as a 3‐min central difference, d/dt(f_o_) is further submitted to wavelet de‐noising, which suppresses unwanted noise while retaining important features. As shown in Figure 3, trend identification, total variation denoising, and wavelet de‐noising successfully reduce irregularity in d/dt(f_o_).

## 4 ALGORITHMS TO SUPPRESS IRREGULARITY IN R: STOCHASTIC DECONVOLUTION WITH A REGULARIZATION PARAMETER AND KALMAN SMOOTHING

**FIGURE 4:**
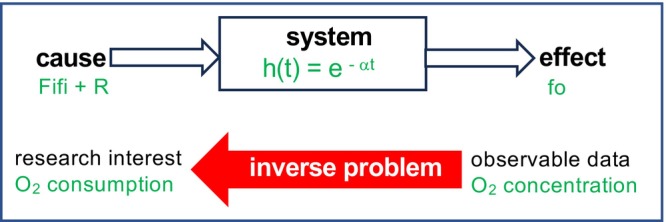
Deconvolution approach as the inverse problem. In whole‐room indirect calorimetry, the gas production rate (R) is estimated from the gas concentration in the chamber. Assuming complete air mixing in the chamber, the system's impulse response as h(t) = e^−αt^ relates O_2_ consumption and O_2_ concentration in the chamber, where α = F_o_/V.

Many signals essential for quantitatively understanding physiologic systems cannot be measured directly in vivo and only their downstream effects can be observed. Instead of following the cause–effect chain, the chain must be reversed to infer the underlying signals of interest. Examples include hormone secretion rates (Sparacino & Cobelli, 1996) or substrate production rates (Nagasaka et al., 1999; Vicini et al., 1997) estimated from the time course of their plasma concentration. In the engineering literature, such problems are referred to as inverse problems or deconvolution (Figure 4).

As a new approach to whole‐room indirect calorimetry, deconvolution with a regularization parameter was introduced. Viewing a whole‐room metabolic chamber as a linear time‐invariant system, it is possible to relate the mass of O_2_ in the chamber at time t and its rate of consumption at τ by a convolution summation. The discrete equation relating O_2_ volume in the chamber (Vf_o_), the sum of O_2_ consumption and incoming O_2_ (R + F_i_f_i_), and the impulse response of the system is shown as follows.

(3) $$V\;f_{0}\left(t\right)=\sum_{\tau =-\infty }^{t}h\left(t-\tau \right)\left\{F_{i}f_{i}h+R\left(\tau \right)\right\}$$

where the function h(*t*) describes the input–output behavior of the chamber and is called the impulse response of the system, and R(τ) is the rate of O_2_ consumption by the subject in the chamber. A part of the right side ($\sum_{\tau =-\infty }^{t}h\left(t-\tau \right)\left(F_{i}f_{i}\right)$) is calculated, transposed to the other side of the equation and expressed in matrix notation as follows.

**FIGURE 5:**
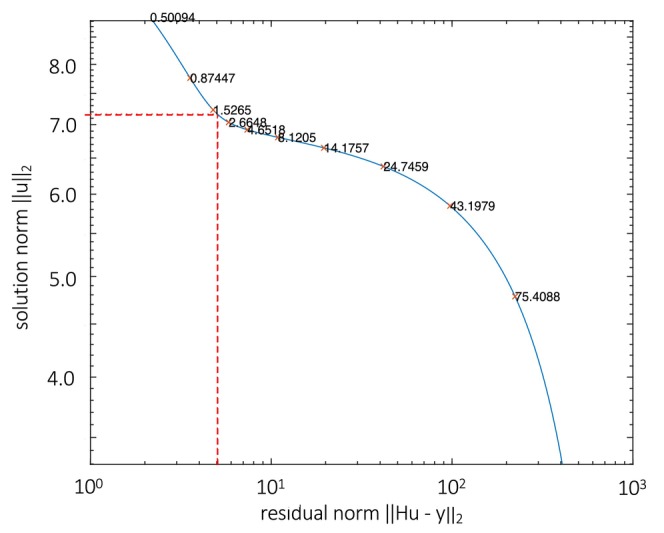
L‐curve method. The L‐curve is a log–log plot of ‖u‖_2_ and ‖Hu − y‖_2_ for all valid regularization parameters γ. The point of maximum curvatures on the L‐curve corresponds to the minimum sum of ‖e‖_2_ and ‖Hu − y‖_2_, and associated γ was selected as the regularization parameter. For this example (sinusoidal signal shown in Figure 7f), γ is estimated to be 1.7583.

(4) y=Hu

where *y* is a vector of dimension n containing the O_2_ volume in the chamber at times *t*_1_ < *t*_2_, …, *t*_n_ (*t*_1_ = 1); u is a vector of dimension n for the O_2_ consumption rate sampled at times 𝜏_1_, 𝜏_2_, …, 𝜏_*n*_ (𝜏_1_ = 0; 𝜏_*i*_ = t_*i*‐1_ for *i* = 2, …, *n*) and assumed to be piecewise constant; and H is an *n* lower triangular matrix of impulse response, whose entries are given analytically by.

(5) $$H_{\mathrm{ij}}=\left\{\begin{array}{cc} 0 & \left(i<j\right)\\ 1 & \left(i=j\right)\\ e^{-\alpha \left(t_{i}-\tau_{i}\right)} & \left(i>j\right) \end{array}\right.$$

where *α* = F/V. Each nonzero element describes the output of the model at time *t*_i_ when all initial conditions are zero and the input is a unit pulse applied between *t*_j−1_ and t_j_. As the direct solution of the deconvolution problem can be ill‐conditioned, the Tikhonov regularization approach (De Nicolao et al., 1997; Vicini et al., 1997) was adopted for whole‐room indirect calorimetry in our original study (Tokuyama et al., 2009), in which the regularized estimate of gas production rate u is defined by nonparametric stochastic deconvolution as

(6) $$\arg\min\left\{\mathrm{SSR}+\gamma u^{T}Q^{T}\mathrm{Qu}\right\}$$

where the first term measures the fidelity to the data as the sum of squared residuals (SSR) in the outgoing air gas concentration, and the second term is introduced to penalize the roughness of the estimate. Q in the second term is an identity matrix, and u^T^Q^T^Qu is the sum of the squared gas production rate. The relative weight given to data fit and solution regularity is governed by the regularization parameter γ. The regularization parameter γ by the discrepancy method is chosen to satisfy Equation 7.

(7) $${\left\|\mathrm{Hu}-y\right\|}_{2}={\left\|e\right\|}_{2}$$

where ‖e‖_2_ is the 2‐norm of the error in the gas concentration measurement. Alternatively, γ is chosen to minimize the sum of the norm of a regularized solution (‖u‖_2_) and the norm of the corresponding residual (‖Hu − y‖_2_) by the L‐curve method (Figure 5). Of note, the L‐curve method does not require a prior estimate of the noise level to determine the regularization parameter γ. While there is clear similarity of this approach (Equation 6) to total variation denoising (Equation 2), there are critical differences between the two approaches, as follows. First, they differ in how roughness is estimated: total variation denoising uses 1‐norm regularization while the deconvolution method uses 2‐norm regularization. Second, total variation denoising (Equation 2) is not dependent on the system's impulse response function (h(t)), that is, total variation denoising is not a deconvolution approach. Consequently, total variation denoising suppresses roughness in d/dt(f_o_), while deconvolution with a regularization parameter suppresses roughness in R, the ultimate target of indirect calorimetry.

Kalman estimation is an algorithm used to estimate non‐observable state variables based on observable variables that may have some measurement error. Adopting this approach, Granato et al. (2004) incorporated variance $\sigma_{u}^{2}$ for the gas production rate in a stochastic model to describe the gas exchange dynamics in a metabolic chamber, which avoids overfitting the non‐observable gas production rate to an observable gas concentration.

## 5 EVALUATION OF ALGORITHMS IN SIMULATIONS

**FIGURE 6:**
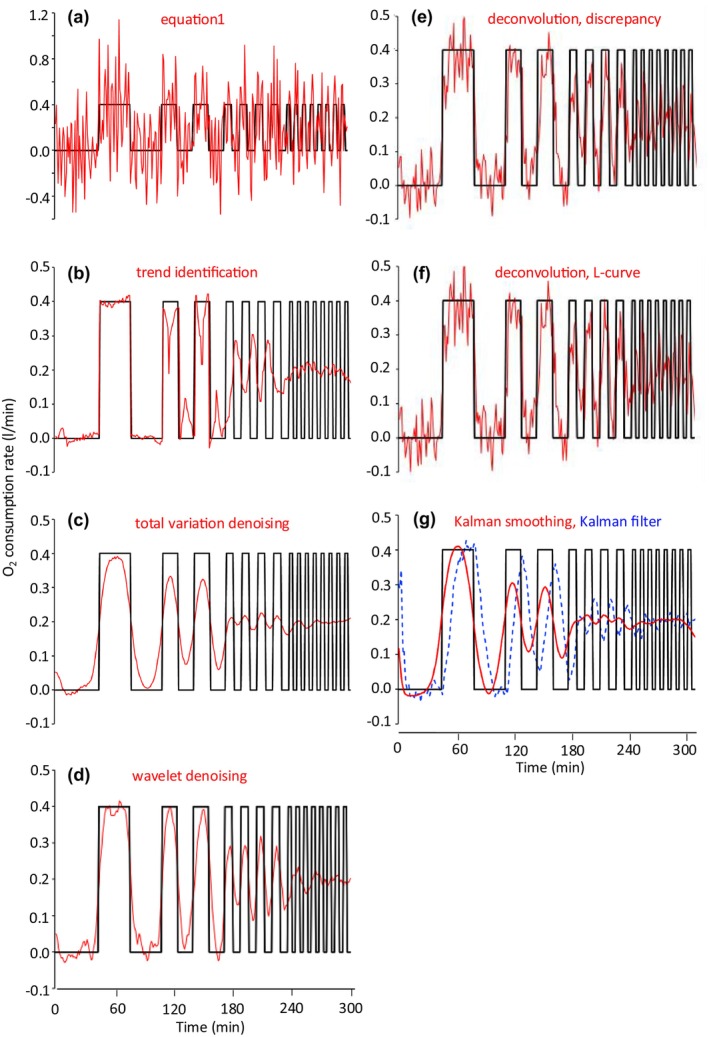
Simulation of O_2_ consumption for intermittent rectangular signals using various algorithms. For simulations, the chamber of the whole‐room calorimeter was modeled as a single compartment (V = 16,626 L), from which air was pulled out at a rate of 62 L/min. The respiratory quotient (RQ) was set at 1.0; F_i_ = F_o_. O_2_ and CO_2_ concentrations of incoming air were 20.93% and 0.03%, respectively. At 45 min into the simulation, O_2_ consumption with a 64‐min period (32 min on, 32 min off) was initiated, followed by period of 32‐, 16‐, and 8‐min durations (**―**). The outgoing air O_2_ concentration was calculated at 1‐min intervals and corrupted with white noise (0.000 ± 0.002%). From the noise‐corrupted gas concentrations, O_2_ consumption rates were estimated (**―**). (a) by Brown's equation (Equation 1), (b) trend identification, (c) total variation denoising, (d) wavelet de‐noising, (e, f) deconvolution with regularization parameters (discrepancy and L‐curve methods), and (g) Kalman smoothing (shown in red) and estimates from the Kalman filter (blue dotted line). To calculate O_2_ consumption rates during the first and last 15 min of the simulation by trend identification, the simulation was extended for 15 min before and after the 300‐min core period. During the additional 15‐min periods, the O_2_ consumption rate was set at 0.00 L/min.

**FIGURE 7:**
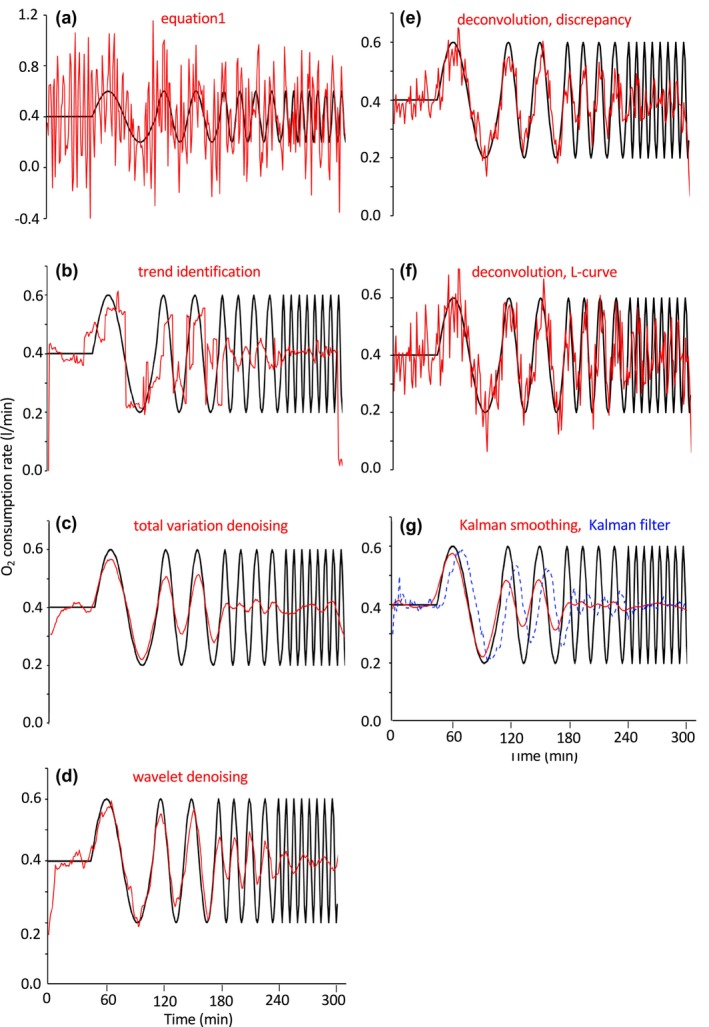
Simulation of O_2_ consumption for sinusoidal signal by various algorithms. For simulations, the chamber of the whole‐room calorimeter was modeled as a single compartment (V = 16,626 L), from which air was pulled out at a rate of 62 L/min. The respiratory quotient (RQ) was set at 1.0; F_i_ = F_o_. O_2_ and CO_2_ concentrations of incoming air were 20.93% and 0.03%, respectively. At 45 min into the simulation, sinusoidal O_2_ consumption patterns with gradually shorter period (64, 32, 16, and 8 min) were applied (**―**). Oxygen concentration in the chamber was calculated at 1‐min intervals and then artificially corrupted with noise (mean ± SD: 0.000 ± 0.002%). From these noise‐corrupted gas concentrations, O_2_ consumption rates were calculated (**―**). (a) by Brown's equation (Equation 1), (b) trend identification, (c) total variation denoising, (d) wavelet de‐noising, (e, f) deconvolution with a regularization parameter (discrepancy and L‐curve methods), and (g) Kalman smoothing (shown in red) and estimates from the Kalman filter (blue dotted line). To calculate O_2_ consumption rates during the first and last 15 min of the simulation by trend identification, the simulation was extended for 15 min before and after the 300‐min core period. During the additional 15‐min periods, the O_2_ consumption rate was set at 0.00 L/min.

The oxygen consumption rate estimated by Equation 1 largely deviated from the true value (Figures 6a and 7a). Trend identification (Henning et al., 1996) improved estimates of O_2_ consumption for rectangular signals with a longer period, such as 64 min and 32 min. On the other hand, the O_2_ consumption rate for signals with a shorter period was out of phase with the true signal (Figures 6b and 7b). These peculiar results were also observed in the CO_2_ appearance rate in an intermittent CO_2_ infusion test (Brychta et al., 2009; Tokuyama et al., 2009). Total variation denoising (Figures 6c and 7c) and wavelet de‐noising (Figures 6d and 7d), which suppress the irregularity in d/dt(f_o_), successfully reduce irregularity in the O_2_ consumption rate, but that for signals of shorter period is overdamped.

**FIGURE 8:**
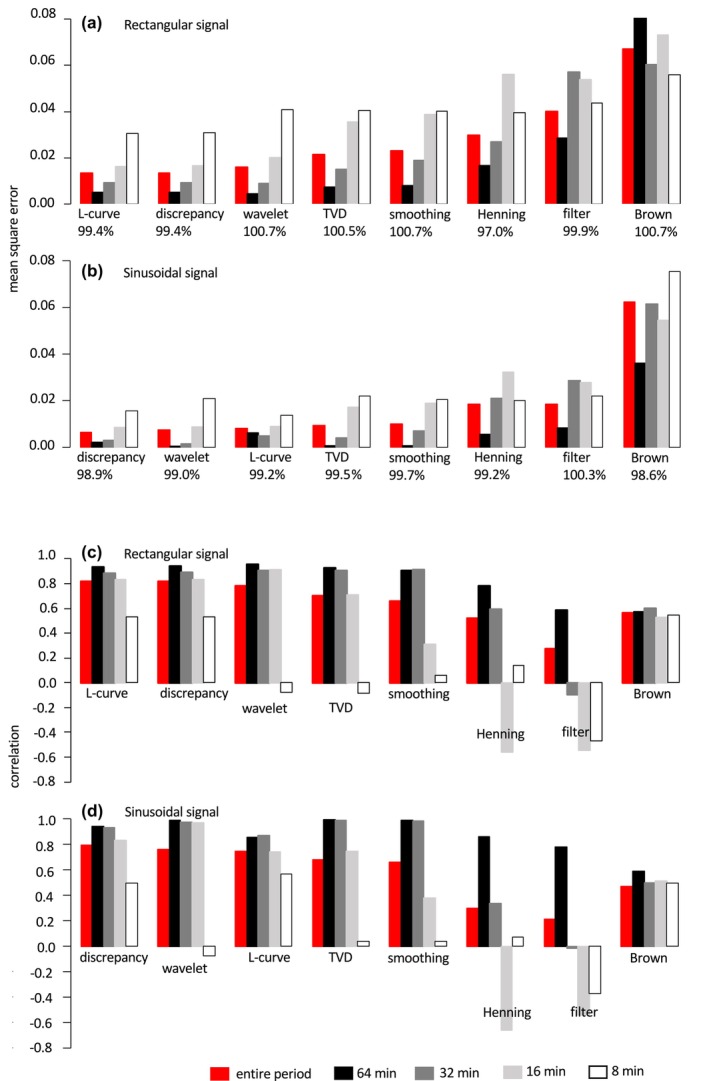
Mean squared error, correlation with the true values, and recovery in simulations. (a, b) Mean squared error for deconvolution with a regularization parameter by the discrepancy method (discrepancy) and L‐curve method (L‐curve), total variation denoising (TVD), wavelet de‐noising (wavelet), Kalman smoothing (smoothing), trend identification (Henning), Kalman filter (filter), and Equation 1 (Brown). Recovery (cumulative O2 consumption over 300 min/true value) is shown under the name of algorithm. Bar graph is color‐coded: ■, red represents the entire simulation period (0–300 min); ■, 64 min period signal (from 45 to 108 min of the simulation); ■, 32 min period signal (from 109 to 172 min); ■, 16 min period signal (from 173 to 236 min); and □, 8 min period signal (from 237 to 300 min). Algorithms are ordered from left to right by increasing mean squared error for the entire simulation period for rectangular and sinusoidal signal simulations, respectively. (c, d) Correlation coefficients relative to the true values.

Both deconvolution with a regularization parameter (Figures 6e,f and 7e,f) and Kalman estimator (filter and smoothing) (Figures 6g and 7g) are stochastic algorithms for estimating the underlying O_2_ consumption rate in the presence of uncertainty, and the theoretical connection between the two algorithms has been examined (De Nicolao et al., 1997). A critical difference between the two algorithms resides in the assumption of variations in the gas production rate. In Kalman estimation, $\sigma_{u}^{2}$ was experimentally determined and subsequently used as a fixed value (2.5 × 10^−3^ l^2^min^−4^), which may not be optimal for another experiment (Granato et al., 2004). On the other hand, γ in the deconvolution approach is regularized for each data set, which may at least partially explain the superior performance of this algorithm over Kalman filtering and Kalman smoothing. Regularization parameter γ in the deconvolution approach was chosen for the discrepancy or L‐curve method in the present study. Performance of the two approaches was similar in simulations with rectangular and sinusoidal signals. Among seven algorithms assessed in the simulations, deconvolution with a regularization parameter resulted in better simulated values in terms of correlation with the true value and the mean squared errors for rectangular and sinusoidal signals. It is noteworthy that the deconvolution approach estimates the O_2_ consumption rate for signals with an 8‐min period (4 min on and 4 min off), which significantly correlates with the true signal (*p* < 0.01) without overfitting (low mean square error). Estimates of other new algorithms were overdamped (total variation denoising, wavelet de‐noising, Kalman smoothing) or negatively correlated with the true value (trend identification, Kalman filter) for an 8‐min period signal (Figure 8). Despite large differences in the mean squared error of the estimate and its correlation with the true value, all algorithms produced similarly good estimates of cumulative O_2_ consumption over 300 min.

The inverse problem is a fundamental topic in data‐driven science and engineering, and numerous algorithms have been developed to solve this problem (Hansen 2008; Hansen, 2010). Our first program of deconvolution with a regularization parameter was written in C++ adopting a subroutine from Numerical Recipes (Press et al., 2002) supplemented with our own source code (Tokuyama et al., 2009). Since 2009, we have used the program run on ordinary desktop or laptop computers, and it takes 1–2 h to complete the calculation. Now, when applying the same principle but running it on the MATLAB platform, the calculation is completed in only 5 s.

## 6 CRITICISM OF DECONVOLUTION WITH A REGULARIZATION PARAMETER AND SIMULATION ANALYSIS

Deconvolution with a regularization parameter has been criticized as impractical, as it requires the calculation of an inverse of a large matrix, with a size equal to the length of the data, or many iterations of a numerical optimization scheme (Pendar et al., 2016). This criticism is based on indirect calorimetry of insects: a grasshopper in a 28‐mL respiratory chamber, with a sampling rate of 10 Hz, that is, 36,000 samples in 1 h. In our human indirect calorimetry, sampling every minute for 24 h adds up to 1440 samples, and the matrix size required for human studies is 1440 × 1440. For human studies, an ordinary laptop computer can complete deconvolution with a regularization parameter. High‐frequency sampling in insect studies requires the manipulation of a 625‐fold larger matrix for deconvolution analysis, which led to the development of additional techniques as hybrid iterative methods for large‐scale inverse problems proposed by Cho et al. (2021).

To the best of our knowledge, the algorithm based on deconvolution for indirect calorimetry has been used by three research groups over the past two decades (Cho et al., 2021; Gribok et al., 2016; Tokuyama et al., 2009), but these algorithms are not the most commonly used algorithms in energy metabolism research. A drawback of our algorithm is that it is inconvenient for other researchers to set up the program. The example set by Cho et al. (2021) prompted us to release the source code on the MATLAB platform.

Before drawing objective conclusions from algorithm comparisons based on simulations, cautious discussion is warranted for two issues. First, the rectangular and sinusoidal signals adopted in our study do not adequately reflect real‐world data situations, except for gas infusion tests. The noise was assumed to be white with an SD of 0.002%, based on 24‐h continuous measurement of standard air. However, the gas concentration in fresh air changes diurnally, and gas mixing kinetics may change depending on the position of the subjects in the chamber. The noise level, signal amplitude (0.4 L/min), flow rate (F), and chamber volume (V) are all directly related to the signal‐to‐noise ratio. In addition, the simulation outcome is also influenced by the duration of simulation (300 min) and the signal period (8, 16, 32, and 64 min). Second, the simulation should be repeated multiple times using different sets of random noise and subjected to rigorous statistical analysis, which was not considered in our original study (Tokuyama et al., 2009) nor in other previous studies (Cho et al., 2021; Granato et al., 2004; Chen et al., 2018; Pendar et al., 2016; Brychta et al., 2009; Henning et al., 1996).

## 7 PHYSIOLOGIC IMPLICATIONS

A standard method for evaluating metabolic flexibility is assessing substrate oxidation during a hyperinsulinemic‐euglycemic clamp. However, this approach does not provide a physiologic context to assess the switching of the oxidized substrate in response to nutrient availability (Carnero et al., 2021). Assessment of dynamic responses of energy metabolism to meals and extended fasting during sleep allows us to evaluate a metabolic flexibility phenotype under physiologic conditions (Gribok et al., 2016; Zhang et al., 2021).

**FIGURE 9:**
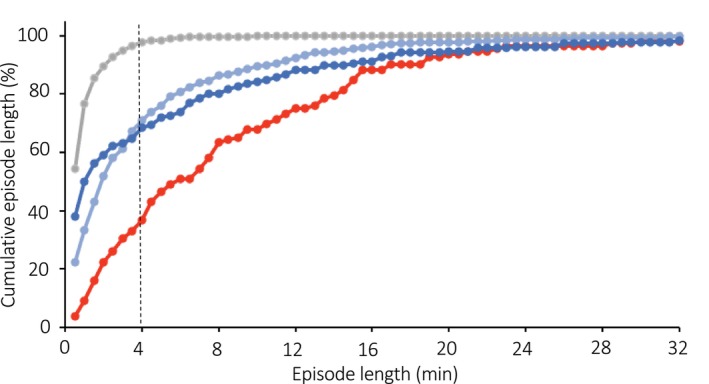
Cumulative distribution of sleep episode length. The cumulative distribution of sleep episode length was calculated for N1 (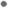), N2 (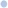), N3 (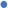), and REM (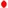) stages. The proportion of episodes longer than 4 min was 2.3%, 29.1%, 31.6%, and 63.4% for N1, N2, N3, and NREM sleep, respectively. Data are unpublished observations for the control condition trial in our previous study (Park et al., 2024).

**FIGURE 10:**
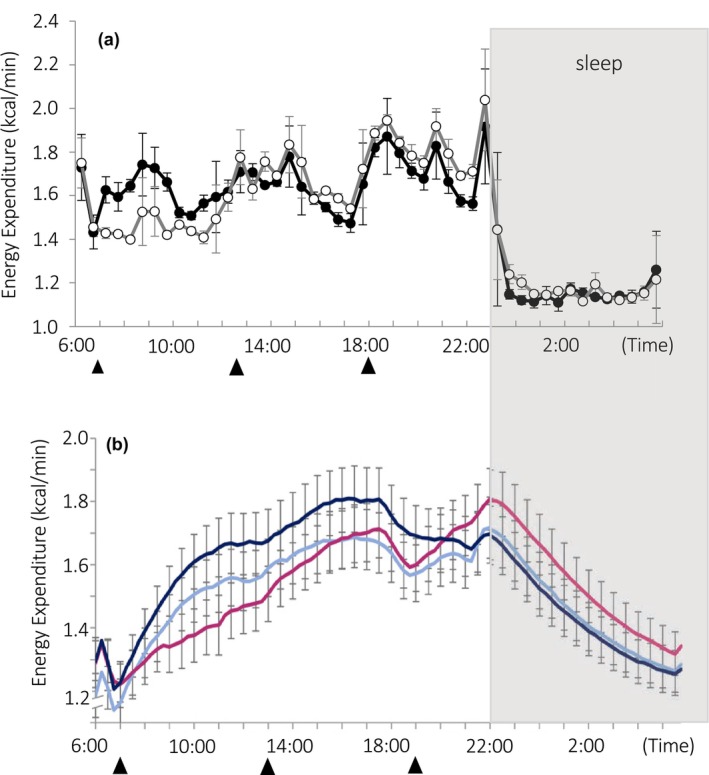
Diurnal changes in energy expenditure. The effect of meal skipping on 24‐h energy expenditure was evaluated in two studies. (a) In our study, energy expenditure was estimated using deconvolution with a regularization parameter (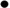 represents data of participants who consumed three meals and 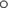 represents those who skipped breakfast) (Ogata et al., 2019). (b) In observation by Nas et al. (2017), the time course appears delayed relative to our results. **―** represents data for participants who consumed three meals, **―** represents those who skipped breakfast, and **―** represents those who skipped the evening meal (Nas et al., 2017). ▲ at the bottom indicates meal times. In both cases, the results were aligned after participants went to bed (‐‐‐‐‐‐). Requests to use previously published figure have been approved (License Number 6066300595380, 6071250932078).

Combining indirect calorimetry with continuous vital sign monitoring is a recent development that favors an improved time resolution of indirect calorimetry. Continuous glucose monitoring systems allow for automatic blood glucose level estimations throughout the day and night. Distinctive meal patterns, such as late evening meals and skipping breakfast, affect diurnal changes in blood glucose and energy metabolism, suggesting a causal link between substrate availability and energy metabolism (Ogata et al., 2019). The time resolution of whole‐room indirect calorimetry has a long way to go to catch up to the time resolution of sleep stage classification from polysomnographic data, in which each 30‐s epoch is annotated as wake, REM, or one of the three NREM stages (Park et al., 2024). However, the amount of time a person remains in a specific stage of sleep without switching to another stage, called the episode length of a sleep stage, is longer than 0.5 min. As shown in Figure 10, 2.3%, 29.1%, 31.6%, and 63.4% of the episodes in N1, N2, N3, and REM sleep are longer than 4 min (Figure 9). In the simulations, changes in O_2_ consumption were detectable by deconvolution with a regularization parameter for an 8‐min period signal: 4 min on and 4 min off (Figures 7, 8). With additional statistical analysis, such as semi‐parametric regression analysis, it is barely possible to detect differences in energy metabolism among sleep stages (Park et al., 2024). Furthermore, individual differences in the time course of sleeping energy metabolism might serve as a window to gain insight into the early‐stage pathogenesis of metabolic inflexibility (Zhang et al., 2021).

**FIGURE A1:**
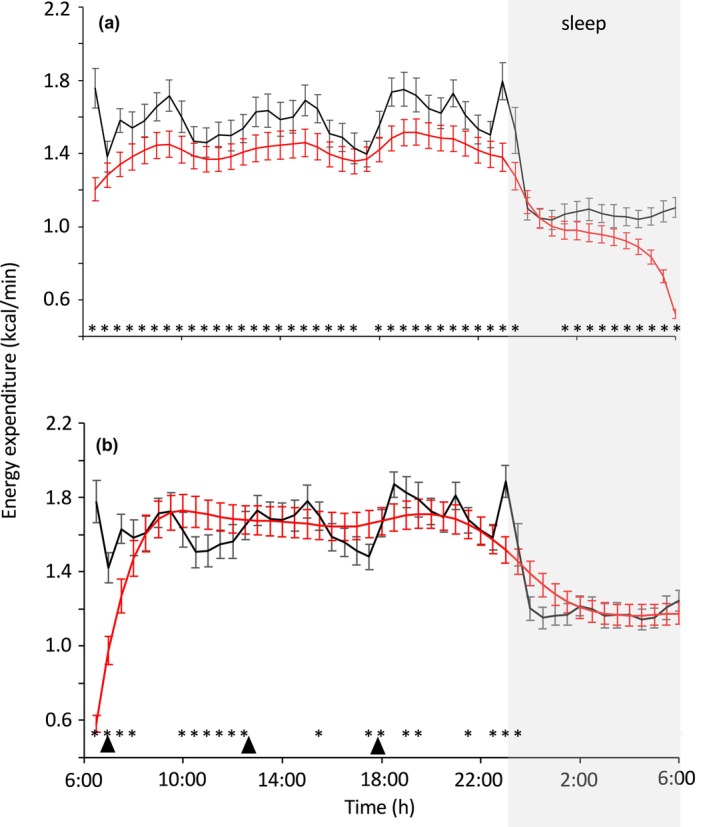
(a) O_2_ consumption and CO_2_ production were calculated by the L‐curve method using optimal γ (2.163 ± 0.063 for VO_2_ and 2.235 ± 0.110 for VCO_2_) and intentionally increased value (γ = 50 for VO_2_ and VCO_2_). (b) O_2_ consumption and CO_2_ production were calculated by the discrepancy method in our previous study, in which the program was written in C++ (Tokuyama et al., 2009). In this algorithm, the roughness of the estimates was penalized by the sum of squared second differences, that is, Q in Equation 6 was the square Toeplitz matrix (*n* × *n*), whose first column is [1, −2, 1, 0, …, 0]^T^. Accordingly, the regularization parameter γ (4610 ± 667) was an order of magnitude greater than that based on the sum of squared gas production rates. In the intentionally overdamped simulation, γ was set at 100,000.

Given that several different algorithms are used for whole‐room indirect calorimetry, we are far from reaching a consensus on the validity of the time course of energy metabolism. This methodologic review was motivated by an apparent difference in the time course of energy metabolism between two studies (Figure 10). It is often assumed that skipping breakfast reduces daily energy expenditure and is therefore a risk factor for obesity. Two studies published in Am J Clin Nutr reported that skipping breakfast does not decrease 24‐h energy expenditure in young subjects if daily caloric intake remains unchanged, with two large meals at lunch and dinner. Although the conclusions regarding the cumulative energy expenditure over 24 h are consistent between the two studies, the immediate decline in energy expenditure after bedtime observed in our study (Ogata et al., 2019) (Figure 10a) was blunted in the other study (Nas et al., 2017) (Figure 10b). An increase in energy expenditure after eating, known as the thermic effect of food, was detected in both studies, but the time course differed: transient increase (Ogata et al., 2019) versus prolonged elevation (Nas et al., 2017). Apparent differences in the time course of the energy expenditure during waking hours and sleep, despite similar experimental protocols, suggest that the energy expenditure profile reported by Nas et al. (2017) is overdamped. A rapid decline in energy expenditure after sleep onset is usually observed in our study (Zhang et al., 2021). In studies by other research groups, energy expenditure decreased by ~30% during the first hour of sleep (Hibi et al., 2017; Jung et al., 2011; Markwald et al., 2013; McHill et al., 2014). In these studies, the O_2_ consumption rate was calculated by trend identification (Hibi et al., 2017) or calculated as O_2_ concentration differences entering and exiting the whole‐room indirect calorimeter (Jung et al., 2011; Markwald et al., 2013; McHill et al., 2014). In another study that did not use whole‐room indirect calorimetry, subjects wore a face mask to collect expired air. The O_2_ consumption rate decreased by 13%~16 % during the first 30 min of sleep, which began 0, 3, or 6 h after subjects' usual bedtime (Fraser et al., 1986). In intentionally overdamped simulations, some features reported by Nas et al. were reproduced, including an ambiguous thermic effect of food and a gradual decline in energy expenditure after bedtime (Appendix Figure A1). Unfortunately, descriptions of methods in the literature that “oxygen consumption and carbon dioxide production were calculated using equations derived by Brown et al.” (Nas et al., 2017) are not sufficient and prevent pinpointing the cause of the apparent discrepancy in the time course of energy metabolism. Of note, all the simulations shown in Figures 7, 8 are based on equations derived by Brown et al. (1984). In general, it is important to adopt an appropriate method when evaluating the temporal relationship between energy metabolism and behaviors such as eating and sleeping, as well as physiologic indices, such as the time course of circulating glucose levels.

## 8 PROPOSAL

Whole‐room indirect calorimetry of humans is time‐consuming, and research may benefit from combining data from many research groups. A similar collaborative approach has already been successfully implemented by a group of researchers using doubly labeled water for indirect calorimetry studies (Pontzer et al., 2021). However, combining apparently contradictory data, as shown in Figure 10, may be misleading. For future collaboration toward advances in calorimetry, it is critical to ensure transparency in the methodologies applied to whole‐room indirect calorimetry.

When we proposed deconvolution with a regularization parameter for whole‐room indirect calorimetry, the performance of the new algorithm was compared against existing algorithms: moving average, trend identification, Kalman filtering, and Kalman smoothing (Tokuyama et al., 2009). This practice has not yet become widely adopted among researchers in this field. In some studies, deconvolution with a regularization parameter is referred to but not compared against the “new” algorithm (Chen et al., 2018; Pendar et al., 2016; Pendar & Socha, 2015). Source code availability has been low. While simulation studies are not expensive, writing the source code required to compare the performance of various algorithms is time‐consuming. As new algorithms for whole‐room indirect calorimetry continue to be proposed, it is important to follow the example set by Cho et al. (2021), who made all MATLAB code and data available from the GitHub website.

## AUTHOR CONTRIBUTIONS

KT conceived of and wrote the manuscript. IP and HO edited and revised the manuscript. KT, HO, and IP wrote MATLAB code and performed simulations.

## FUNDING INFORMATION

No funding.

## CONFLICT OF INTEREST STATEMENT

All authors have no conflict of interests.

## ETHICS STATEMENT

No ethical issue due to simulation study.

## Data Availability

Total variation denoising (Chen et al., 2018) was performed using a MATLAB code by Selenick (2017). MATLAB code for Wavelet de‐noising was provided by Brychta et al. (2009). MATLAB code for discrepancy and l‐curve method for deconvolution with regularization parameter is listed in GitHub (https://github.com/Tokuyama693/Deconvolution‐for‐Metabolic‐Chamber).

## Intentionally overdamped estimation of energy expenditure

Energy expenditure for the three‐meal condition in our previous study (Ogata et al., 2019) was calculated from O_2_ consumption, CO_2_ production, and urinary nitrogen excretion (6.82 ± 0.81 mg/min). Compared to the estimation by optimal γ (**―**), estimation with larger γ (**―**) resulted in an ambiguous thermic effect of food and a slower decline in energy expenditure during the first hour after bedtime. Values are mean ± SE of 10 subjects. The asterisks in each panel represent a statistically significant difference by a 2‐factor repeated measures analysis of variance (ANOVA) (Figure A1).
